# Percutaneous transcatheter occlusion to treat incomplete surgical left atrial appendage closure

**DOI:** 10.1016/j.hrcr.2025.12.008

**Published:** 2025-12-25

**Authors:** Zachary Ostreicher, Nicholas Beccarino, Kayla Levy, James K. Gabriels, Stuart Beldner

**Affiliations:** Cardiovascular Institute, North Shore University Hospital, Northwell, New Hyde Park, New York

**Keywords:** Atrial fibrillation, Left atrial appendage occlusion, Amulet, Transcatheter occlusion, Surgical closure

**Key Teaching Points**

▪ Incomplete surgical closure of the left atrial appendage has been reported in up to one-third of cases and is associated with an increased thromboembolic risk.
▪ Incomplete surgical left atrial appendage closure complicates future attempts at percutaneous occlusion.
▪ Owing to its lobe and disk design, the Amplatzer Amulet device may be safely used in anatomically complex or surgically altered left atrial appendage remnants.

## Introduction

Surgical left atrial appendage closure (S-LAAC) is commonly performed during cardiac surgery to reduce the risk of thromboembolic events in patients with atrial fibrillation (AF). Incomplete closure has been reported in approximately one-third of cases, depending on the surgical technique used: suture ligation, stapling, or clipping.^1^ Any incomplete closure may paradoxically increase the risk of stroke.^1^ In patients who have indications for left atrial appendage (LAA) occlusion, previous incomplete surgical closure complicates percutaneous occlusion. Several percutaneous strategies have been explored in these patients. Previous reports describe the use of the Amplatzer vascular plug II and III, coiling, and the Watchman FLX, which have shown promising outcomes in multicenter series.^2^, ^3^, ^4^ A single case report has reported that the Amplatzer Amulet device can be used in patients with incomplete S-LAAC.^5^ We describe 2 cases in which the Amplatzer Amulet device was used to safely and effectively treat patients with residual flow after incomplete S-LAAC.

## Case reports

The first patient was a 72-year-old man with a history of hypertension, hyperlipidemia, bioprosthetic mitral and aortic valve replacement in 2005, dual-chamber pacemaker in 2016 for tachy-brady syndrome, heart failure, and persistent AF despite multiple previous ablations. In 2017, he underwent repeat bioprosthetic aortic and mitral valve replacement for prosthetic valve dysfunction in the setting of pannus formation, at which time his appendage was oversewn. The patient recently experienced repeated mechanical falls with trauma and was felt to be at high risk of long-term anticoagulation. He presented to our institution for percutaneous LAA remnant occlusion in this setting.

The second patient was an 85-year-old woman with hypertension, embolic stroke, paroxysmal AF, dual-chamber pacemaker implantation for tachy-brady syndrome in 2012, and hypertrophic subaortic aortic stenosis for which she underwent septal myomectomy and bioprosthetic mitral and aortic valve replacement in 2014. Similar to the first case, she had received incomplete S-LAAC during her previous procedure. She had recently developed recurrent gastrointestinal and genitourinary bleeding requiring multiple transfusions and precluding continued anticoagulation. She was also referred to our institution for percutaneous occlusion of the remaining LAA. Preprocedural computed tomography imaging in both patients demonstrated incomplete LAA closure (LAAC) with narrow residual necks, measuring approximately 6 mm in width.

**Figure 1:**
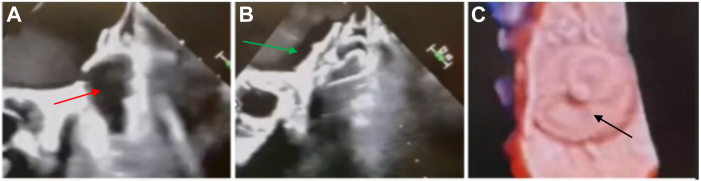
Case :1 incomplete surgical LAA closure successfully occluded with Amplatzer Amulet (22 mm). **A:** Preprocedural TEE shows incomplete surgical LAA closure with a narrow residual neck and persistent flow from the left atrium into the LAA remnant (*red arrow*). **B:** After transseptal access and delivery of a 22 mm Amplatzer Amulet, the lobe is seated in the remnant and the disk (*green arrow*) covers the atrial opening. Color Doppler demonstrated no peridevice leak, confirming immediate occlusion. **C:** 3-dimensional TEE confirms a well-seated device (*black arrow*) with complete coverage of the residual neck and stable device position. LAA = left atrial appendage; TEE = transesophageal echocardiogram.

**Figure 2:**
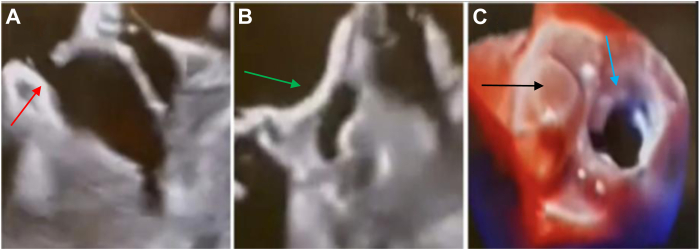
Incomplete surgical LAA closure successfully occluded with Amplatzer Amulet (25 mm). **A:** Preprocedural TEE demonstrates residual communication through a narrow surgical fenestration (*red arrow*) after previous surgical LAA closure. **B:** After deployment of a 25 mm Amplatzer Amulet (*green arrow*), TEE shows complete occlusion of the LAA without residual color Doppler flow. **C:** 3-dimensional TEE at follow-up demonstrates a well-seated device with full coverage of the ostium (*black arrow*), consistent with durable occlusion. The mitral valve can be seen (*blue arrow*). LAA = left atrial appendage; TEE = transesophageal echocardiogram.

Both procedures were performed under transesophageal echocardiographic (TEE) guidance, which confirmed the incomplete LAACs and narrow neck morphology (Figures 1A and 2A). Closure methods including detachable coils, vascular plugs, and radiofrequency ablation were considered. However, given the large cavity and the need for proven thromboembolic protection, the Amulet was thought to provide the best balance of safety, coverage, and long-term durability. In the first case, TEE demonstrated a small, shallow appendage remnant with a narrow neck, which guided the selection of a 22 mm Amulet to ensure adequate seal without excessive protrusion into the atrium. In the second case, the remnant cavity was slightly larger and deeper, leading to the selection of a 25 mm Amulet. After achieving transseptal access, an Amplatzer TorqVue 45° × 45° sheath was advanced into the left atrium. The LAA was accessed using a Guideright wire, over which a pigtail catheter was advanced through the surgical fenestration in both patients. Contrast injection confirmed persistent communication with the LAA through narrow necks (Figures 1B and 2B).

Using the pigtail catheter as a rail, the sheath was advanced into the appendage. A 22 mm Amplatzer Amulet was selected for the first case and a 25 mm device for the second. After deployment, complete closure was observed on TEE, with no peridevice leak in either case (Figures 1C and 2C). Both patients tolerated the procedure well without complications. Follow-up imaging at 90 days (TEE for the first patient and computed tomography for the second) showed well-seated devices with no residual leak, and anticoagulation was safely deescalated.

These cases illustrate the effectiveness of the Amplatzer Amulet in addressing incomplete S-LAAC, which can paradoxically increase thromboembolic risk. The dual-seal design of the Amulet device makes it a strong option for sealing narrow-necked remnants, especially in patients who are poor candidates for long-term anticoagulation.

## Discussion

Incomplete surgical closure of the LAA presents a clinical challenge owing to its association with residual stroke risk and anatomic distortion, complicating percutaneous occlusion.^1^ When surgical closure fails, percutaneous methods offer a minimally invasive approach to complete occlusion in patients who are unable to tolerate anticoagulation. Several strategies have been reported in this setting. The Amplatzer vascular plug has been successfully employed in cases of residual leaks after attempts at S-LAAC in several cases.^2^^,^^3^ The Watchman FLX has also been used in this setting with safety and efficacy shown in small multicenter case series involving 3 patients.^4^ Coils have also been used to close small residual leaks after surgical or device-based LAAC, with success rates of >90% in larger series of 30 patients.^6^ Although all of these options were available during our cases if needed, the Amulet was chosen owing to its robust clinical data for stroke prevention and LAA occlusion, including in anatomically complex or surgically altered appendages. Based on the size and morphology of the residual ostia, the Amulet’s lobe and disk design seemed ideally suited to achieve complete coverage and stable anchoring in these cases. The Amplatzer Amulet device is emerging as an alternative method of closure for anatomically complex LAAs. Initial experiences with the Amulet device in de novo appendages revealed noninferiority regarding safety and efficacy for stroke prevention compared with the Watchman device, but superiority for LAA occlusion at the expense of slightly higher procedural complications.^7^ This is likely because of the lobe and disk design of the Amulet, which allows the device to conform to and effectively occlude a wide variety of LAA anatomies, making it an ideal choice in cases that pose anatomic challenges. These features also make this device uniquely well suited to cases of incomplete S-LAAC. With the lobe positioned in the appendage body, the disk is able to completely cover any residual narrow opening, allowing for a complete seal with stable positioning. To the best of our knowledge, we report 2 of the earliest multicase descriptions demonstrating successful percutaneous occlusion of incomplete S-LAAC using the Amulet. Our cases align with the single previous experience, demonstrating safe and effective closure of surgically altered appendages.^5^ These findings reinforce the Amulet’s utility as an important tool in the management of incomplete S-LAAC.

The limitations of this technique include the potential difficulty in advancing catheters or wires through a distorted or narrowed appendage remnant. In 1 of our previous cases, there was difficulty advancing the pigtail catheter over the wire into the LAA. In such scenarios, a useful workaround is to first advance a Terumo wire into the remnant and then telescope a Terumo Glidecath over it. Once positioned, a stiffer wire such as the Amplatz Superstiff can be advanced through the Glidecath to provide adequate support for delivery systems. Another important consideration is the need for flexibility in closure strategy. In 1 case, the initial plan to use a vascular plug was revised during the procedure in favor of detachable coils owing to challenging anatomy, which led to a successful result. This highlights the importance of having multiple closure tools available in the room to accommodate bail-out strategies. Finally, coil-based closure often requires more than 1 coil to achieve a complete seal. In the previously mentioned case series, the median number of coils used per case was 3, with an interquartile range of 2–4.^6^ This emphasizes the need for preprocedural planning and adequate inventory preparation.

## Conclusion

The Amplatzer Amulet device represents a safe and effective option for percutaneous closure of the LAA in cases where surgical closure is incomplete. The lobe and disk design facilitates secure sealing even in anatomically complex or surgically altered appendages.

## Disclosures

The authors have no conflicts of interest to disclose.
